# Shotgun Metagenomics Reveals the Benthic Microbial Community Response to Plastic and Bioplastic in a Coastal Marine Environment

**DOI:** 10.3389/fmicb.2019.01252

**Published:** 2019-06-07

**Authors:** Lee J. Pinnell, Jeffrey W. Turner

**Affiliations:** Department of Life Sciences, Texas A&M University – Corpus Christi, Corpus Christi, TX, United States

**Keywords:** plastic, bioplastic, biodegradation, sulfur cycling, coastal sediments, metagenomics

## Abstract

Plastic is incredibly abundant in marine environments but little is known about its effects on benthic microbiota and biogeochemical cycling. This study reports the shotgun metagenomic sequencing of biofilms fouling plastic and bioplastic microcosms staged at the sediment–water interface of a coastal lagoon. Community composition analysis revealed that plastic biofilms were indistinguishable in comparison to a ceramic biofilm control. By contrast, bioplastic biofilms were distinct and dominated by sulfate-reducing microorganisms (SRM). Analysis of bioplastic gene pools revealed the enrichment of esterases, depolymerases, adenylyl sulfate reductases (*aprBA*), and dissimilatory sulfite reductases (*dsrAB*). The nearly 20-fold enrichment of a phylogenetically diverse polyhydroxybutyrate (PHB) depolymerase suggests this gene was distributed across a mixed microbial assemblage. The metagenomic reconstruction of genomes identified novel species of *Desulfovibrio, Desulfobacteraceae*, and *Desulfobulbaceae* among the abundant SRM, and these genomes contained genes integral to both bioplastic degradation and sulfate reduction. Findings indicate that bioplastic promoted a rapid and significant shift in benthic microbial diversity and gene pools, selecting for microbes that participate in bioplastic degradation and sulfate reduction. If plastic pollution is traded for bioplastic pollution and sedimentary inputs are large, the microbial response could unintentionally affect benthic biogeochemical activities through the stimulation of sulfate reducers.

## Introduction

Microorganisms rapidly colonize and form biofilms on biotic and abiotic surfaces in marine environments (Dang and Lovell, 2016). As such, marine microbial communities are commonly distinguished as free-living or particle-associated (DeLong et al., 1993; Crump et al., 1999; Hollibaugh et al., 2000). A particle-associated lifestyle is thought to provide microorganisms with increased nutritional resources and environmental stability (Azam et al., 1994). Detrital aggregates, in particular, are hot spots of microbial diversity that are fundamentally distinct in comparison to free-living communities (DeLong et al., 1993; Crump et al., 1999). Particles are also habitats of enhanced enzyme activity (Karner and Herndl, 1992; Kellogg and Deming, 2014) that contribute to the rapid turnover of particulate organic matter (Biddanda and Pomeroy, 1988; Azam and Long, 2001) and it is clear that particle-associated microorganisms play an important role in biogeochemical processes (Ploug et al., 1999; Seymour et al., 2017).

Plastic pollution has introduced a new surface for microbial colonization and biofilm formation (Zettler et al., 2013; Oberbeckmann et al., 2014). A recent global assessment of all mass-produced plastics estimated that 4.9 billion tons of plastic waste was discarded in landfills or natural environments (Geyer et al., 2017). Oceans serve as a sink for said waste with an estimated 4.8–12.7 million metric tons entering the oceans annually (Jambeck et al., 2015; Geyer et al., 2017). It is clear that marine microorganisms colonize and form biofilms on floating plastic debris and it is also clear that plastic-associated microbial communities are distinct compared to free-living communities (Bryant et al., 2016; Oberbeckmann et al., 2016).

Several studies have characterized pelagic microbe-plastic interactions (Carson et al., 2013; Zettler et al., 2013; Bryant et al., 2016) but relatively few studies have characterized microbe-plastic interactions in benthic systems (Harrison et al., 2014; De Tender et al., 2015; Nauendorf et al., 2016). In the North Pacific Gyre, a previous study reported that microplastics were colonized by diatoms (Carson et al., 2013) and a separate study reported an increased concentration of Chl *a* and an increased abundance of nitrogenase genes in microplastic biofilms (Bryant et al., 2016). By contrast, understanding of benthic microbe-plastic interactions is limited. The transport of plastic debris to benthic systems is facilitated by sinking; debris with a density higher than seawater (1.02 g cm^−3^) sinks immediately while floating debris loses buoyancy with biofouling (Barnes et al., 2009; Andrady, 2011). Sinking is therefore an important process and benthic systems are sinks for plastic debris accumulation (Thompson et al., 2004; Van Cauwenberghe et al., 2015).

In coastal regions, benthic microorganisms support primary producers in the overlying water column by the remineralization of organic-rich debris (Klump and Martens, 1981). The remineralization of carbon in anoxic coastal sediments is predominantly carried out *via* sulfate reduction (Jørgensen, 1977). The microbial pathway for dissimilatory sulfate reduction involves the reduction of sulfate to sulfite by sulfate adenyltransferases (Sat) and adenylyl-sulfate reductases (AprBA), followed by the reduction of sulfite by sulfite reductases (DsrAB) (Anantharaman et al., 2018). Climate warming and eutrophication are expanding the distribution of anoxic sediments worldwide (Vigneron et al., 2018) and recent studies have clearly shown that sulfate and sulfite reducers are more abundant, diverse, and widespread than previously believed (Müller et al., 2014; Anantharaman et al., 2018; Hausmann et al., 2018; Vigneron et al., 2018; Zecchin et al., 2018). Yet, the effect plastic pollution may have on sulfate-reducing microorganisms (SRM) is an open question.

In 2018, the United Nations Environmental Program reported that more than 60 countries approved bans or levies on single-use plastics (UNEP, 2018). In the same year, the European Parliament approved a ban on the top 10 single-use plastics and called for a reduction in single-use plastics (European Commission, 2018). The proposal states “this systemic change and material substitution will also promote bio-based alternatives and an innovative bioeconomy.” In the wake of legislative actions and increased public awareness, the production of bioplastics such as polyhydroxyalkanoate (PHA) is predicted to increase tenfold (Aeschelmann and Carus, 2015). If bioplastic is similarly discarded, the subsequent increase in bioplastic waste entering the oceans will introduce yet another surface for microbial colonization; and, while some research has investigated the response of marine organisms to bioplastic (Eich et al., 2015; Pauli et al., 2017), little is known about how microorganisms respond to bioplastic versus petroleum-based plastic.

The aim of this study was to characterize the microbial communities and individuals that form biofilms on plastic (polyethylene terephthalate; PET) and bioplastic (PHA) in a coastal benthic habitat. For comparative purposes, ceramic pellets were included as a biofilm control. This study was primarily designed to address two questions. Does the introduction of either plastic or bioplastic select for a distinct microbial community compared to a biofilm control, and does this introduction promote a significant shift in taxa or enzyme pools with implications for polymer degradation or biogeochemical cycling? We hypothesized that the PET biofilms would be indistinguishable from the ceramic biofilms whereas the introduction of PHA would select for the growth of a distinct microbial assemblage involved in polymer degradation and biogeochemical cycling.

## Materials and Methods

### Site Description

The Laguna Madre (Texas, USA) is a large coastal lagoon in the northern Gulf of Mexico (NGOM). The lagoon is divided into two sections: Upper Laguna Madre (ULM) and Lower Laguna Madre (LLM). The microcosms described in this study were deployed adjacent to a dredge material island at 27°32′39.0“N and 97°17’07.7”W in the ULM (Supplementary Figure S1).The ULM is 76 km in length, 6 km in width, has an average depth of 0.8 m, and is separated from the NGOM by the Padre Island National Seashore (Tunnell, 2002). The dredge material island itself is remote to the nearest population center (Corpus Christi, TX) and is therefore relatively free of plastic pollution.

### Sample Deployment and Collection

A microcosm was designed to circumvent the challenges of isolating preexisting plastic debris from heterogeneous sediment samples. A microcosm also permitted the inclusion of a ceramic biofilm control. Briefly, 6.0 g of ceramic pellets (Lyman Products, Middletown, CT, USA), 3.0 g of PET pellets (M&G Chemicals, Ettelbruck, Luxembourg), and 3.0 g of PHA pellets (Doctors Foster and Smith, Rhinelander, WI, USA) were deployed in triplicate inside custom-made microbial capsules (MicroCaps; Supplementary Figure S2) at the sediment–water interface for 28 days (May 24, 2016 to June 21, 2016). All pellets were approximately 3–4 mm in diameter and, therefore, the PET and PHA pellets were considered microplastics (Andrady, 2011). MicroCaps utilized 315-μm Nitex mesh to contain the pellets and permit the exchange of water, nutrients, bacteria, and some grazers but limit the entry of larger organisms. The microcosms and triplicate 1-L seawater samples (also from the sediment–water interface) were collected at the end of the exposure. All samples were stored on ice and processed immediately upon return to the laboratory, with the extraction procedure starting within 2 h of sample collection. Substrates were washed three times with 25 ml of 0.22-μm filter-sterilized, site-specific seawater to remove any organisms not part of the biofilm. Seawater (100 ml) was pre-filtered through 315-μm Nitex mesh and the microbial community was collected by membrane filtration on a 0.22-μm polycarbonate filter (Millipore Sigma, Burlington, MA, USA) in triplicate. Environmental parameters from the start and end of the exposure period were collected using a 6,920 V2–2 Multi-Parameter Water Quality Sonde (YSI, Yellow Springs, OH, USA) and varied minimally as follows: water temperature (28.56–29.28°C), salinity (37.78–40.28), pH (8.35–8.47), and DO (8.30–5.68 mg L^−1^).

### DNA Isolation

Genomic DNA was isolated in triplicate for each sample type (seawater, ceramic, PET, and PHA) using a modified version of a high-salt and sodium dodecyl sulfate-based method (Zhou et al., 1996). The only modification from the original procedure was that instead of 5.0 g of soil, DNA was isolated from a quartered 0.22-μm polycarbonate filter (seawater), 6.0 g of ceramic pellets, 3.0 g of PET, or 3.0 g PHA pellets. Due to their higher density, 6.0 g of ceramic was approximately equal in volume to 3.0 g of either plastic type. Following isolation, DNA was quantified (ng μl^−1^) and assayed for quality (A_260_/A_280_ and A_260_/A_230_) using a BioPhotometer D30 (Eppendorf, Hamburg, Germany) and the “ds_DNA” methods group with default settings. Final DNA concentrations were verified using a Qubit Fluorometer (Thermo Fisher Scientific, Waltham, MA, USA). The DNA was stored in the dark at −20°C prior to sequencing.

### Metagenome Sequencing

Metagenomic library preparation and sequencing were carried out by Molecular Research LP (Shallowater, TX, USA). A total of 12 metagenomes were sequenced: three seawater, three ceramic, three PET, and three PHA. Libraries were prepared using the Nextera XT DNA Library Preparation Kit (Illumina Inc., San Diego, CA, USA) and diluted to 10.0 pM. The average library size was determined using an Agilent 2,100 Bioanalyzer (Agilent Technologies, Santa Clara, CA, USA). Sequencing was performed on the Illumina HiSeq 2,500 platform using 2×150 bp paired-end read chemistry. The DNA concentration (ng μl^−1^) and average size (bp) of the sequencing libraries and the number of sequence reads produced are reported in Supplementary Table S1. Raw sequence reads were submitted to the European Nucleotide Archive and the European Bioinformatics Institute’s (EBI) Metagenomics Pipeline (Mitchell et al., 2016) version 3.0, which includes an automated workflow for read processing. Briefly, overlapping reads were merged with SeqPrep (John, 2011), low-quality reads and adapter sequences were trimmed using trimmomatic (Bolger et al., 2014), and reads less than 100 bp were removed using BioPython (Cock et al., 2009). Identification of the 16S rRNA reads was performed using rRNASelector (Lee et al., 2011) and FragGeneScan (Rho et al., 2010) was used to find reads containing predicted coding sequences (pCDS) greater than 60 nucleotides in length. The number of sequenced reads ranged from 26,860,030 to 36,522,626, with an average of 30,888,258 reads per sample. Following quality control and merging, the number of remaining reads ranged from 11,273,807 to 22,918,149, with an average of 16,270,447 processed reads per sample.

### Microbial Community Composition

Operational taxonomic units (OTUs) were assigned using the QIIME (Caporaso et al., 2010) version 1.9 closed-reference OTU picking protocol and the SILVA 128 SSU 97% database (Quast et al., 2013) with reverse strand matching enabled. Beta-diversity was analyzed using weighted UniFrac (Lozupone et al., 2011) values calculated in QIIME. Permutational Multivariate Analysis of Variance (PERMANOVA) was used to test for significant differences between communities using Primer 7 with the PERMANOVA+ package (Clarke and Gorley, 2015) (PRIMER-E Ltd., Plymouth, UK). PERMANOVA was performed using 999 permutations based on the weighted UniFrac from the beta-diversity analysis in QIIME. Due to the low number of unique permutations possible for pairwise tests, Monte Carlo simulations were used to generate *p*’s for all pairwise comparisons.

### Enrichment of Alpha/Beta Hydrolases

Predicted coding sequences (pCDS) from each of the 12 metagenomes were aligned against a modified database^1^ of protein sequences from the ESTHER database of alpha/beta hydrolases (Hotelier et al., 2004). Proteins included in the modified database were restricted to depolymerases, esterases, lipases, and cutinases as these were previously implicated in plastic-microbe interactions (Yoshida et al., 2016). To reduce redundancy within the database, CD-HIT (Clarke and Gorley, 2015) version 4.7 was used to cluster protein sequences with default settings. The modified database used here included 10 protein families containing a total of 1,079 protein sequences. Alignments were performed using the command-line version of blastp, as implemented in BLAST+ (Camacho et al., 2009) version 2.6.0, with an expect value (*e*) cutoff of 10^−5^, a minimum alignment length of 30 amino acids, and a minimum percent identity of 50%. To visualize the effect of sample type on hydrolase profiles, proportions of positive alignments were normalized to the total number of positive alignments per sample, and a hierarchical cluster analysis based on a Bray-Curtis resemblance matrix was performed. To test for significant differences among profiles, a similarity profile analysis (SIMPROF) (Clarke et al., 2008) using 999 permutations and a significance level of 0.05 was performed in Primer 7. Additionally, the abundance of protein sequences was normalized to the total pCDS and compared between all samples (*n* = 12).

### Relatedness of Polyhydroxybutyrate Depolymerases

Processed reads for each of the PHA samples (*n* = 3) were co-assembled using MEGAHIT (Li et al., 2015) version 1.1.1. Co-assembly was performed using the “meta-large” preset for large and complex communities, and MEGAHIT was called as follows:

megahit –presets meta-large -r input --min-contig-len 1,000 -o output -t 40.

To recover the coding sequences of a significantly enriched polyhydroxybutyrate (PHB) depolymerase (identified using the hydrolase database above), the representative PHB depolymerase gene sequence was aligned to the 118,520 co-assembled contigs using tblastn with an *e* cutoff of 10^−5^, a minimum alignment length of 100 bp, and a minimum percent identity of 50%. An alignment to NCBI’s RefSeq genomic database was carried out with the same parameters in an effort to include gene sequences from previously described organisms. The relatedness of the 46 PHB depolymerase gene sequences from this study and gene sequences from the top 10 most similar genera from RefSeq was inferred by constructing a maximum-likelihood (ML) tree with IQ-TREE (Nguyen et al., 2015) version 1.6.1. Sequences were aligned using the M-Coffee mode of T-Coffee (Notredame et al., 2000) that combines results from eight different aligners and the tree was generated with 1,000 ultrafast bootstraps (Minh et al., 2013) using the best-fit model (TPM3u + F + R3) as determined by ModelFinder (Kalyaanamoorthy et al., 2017). The phylogenetic tree was annotated with FigTree version 1.4.3^2^. Sequence similarity between the 46 PHB depolymerase gene sequences was determined using trimAl (Capella-Gutiérrez et al., 2009) version 1.4.15.

### Recovery and Analysis of Genomes of Sulfate-Reducing Microorganisms

To characterize individual SRM within the PHA biofilm community, metagenome-assembled genomes (MAGs) were recovered using a previously described method (Parks et al., 2017). Briefly, the processed reads were mapped to the co-assembled PHA metagenomic contigs (produced using MEGAHIT above) with BWA (Li and Durbin, 2010) version 0.7.15-r1142 using the BWA-MEM algorithm with default parameters. Genomes were recovered with MetaBAT (Kang et al., 2015) version 2.12.1 using default MetaBat2 settings and a minimum contig size of 2000 bp. The resulting bins were merged, filtered, and refined using CheckM (Parks et al., 2015) version 1.0.11 and RefineM version 0.0.23, as described previously (Parks et al., 2017). Of the 46 bins produced, six MAGs were retained for further exploration based on criteria adopted by Parks et al. (Parks et al., 2017): an estimated quality of ≥50 (completeness – 5× contamination), scaffolds resulting in an N50 ≥ 10 kb, containing <100 kb ambiguous bases, and consisting of <1,000 contigs and < 500 scaffolds. Of those six MAGs, the three belonging to SRM as inferred with the “tree_qa” function in CheckM were explored further.

The three SRM MAGs were analyzed with the Pathosystems Resource Integration Center’s (PATRIC) (Wattam et al., 2017) comprehensive genome analysis service. The PATRIC database contains over 190,000 bacterial genomes and has been increasingly used in environmental studies (Harke et al., 2015; Skennerton et al., 2016; Ortiz-Álvarez et al., 2018).The automated PATRIC service includes annotation with RASTtk (Brettin et al., 2015), prediction of nearest neighbors with Mash/MinHash (Ondov et al., 2016), clustering of homologous proteins with OrthoMCL (Enright et al., 2002), alignment of conserved clusters with MUSCLE (Edgar, 2004), trimming with Gblocks (Talavera and Castresana, 2007), and concatenation followed by inference of a ML tree with RAxML (Stamatakis, 2014). All complete *Desulfovibrionaceae, Desulfobacteraceae*, and *Desulfobulbaceae* genomes in the PATRIC database (*n* = 43, 21, and 11, respectively) were included in the analysis. The resulting tree was annotated using FigTree version 1.4.3. Subsequently, these three MAGs were compared to all publicly available *Desulfovibrionaceae, Desulfobacteraceae*, and *Desulfobulbaceae* genomes in GenBank (*n* = 122, 107, and 65, respectively) by average nucleotide identity (ANI) using fastANI (Jain et al., 2018), using >95% ANI as the intraspecies threshold and <83% as an interspecies threshold. Additionally, PATRIC’s “Protein Family Sorter” tool was used to identify sulfate-reducing proteins within the three SRM MAGs. To identify any polymer degradation protein sequences within the MAGs, pCDS were generated using Prodigal (Hyatt et al., 2010) version 2.6.3 and were aligned against the custom hydrolase database (described above) using the same search parameters.

### Dissimilatory Sulfur Reduction Potential

Predicted coding sequences (pCDS) from each of the 12 metagenomes were aligned against three databases representing the three steps of the dissimilatory sulfate reduction pathway. The database for the first step of the pathway (reduction of sulfate to adenylyl sulfate [APS]) included all sulfate adenylyltransferase (SAT/MET3) protein sequences in the UniProtKB protein database. The database for the second step of the pathway (reduction of APS to sulfite) included all APS reductase (AprBA) protein sequences in the UniProtKB protein database. To address redundancy within both databases, CD-HIT (Clarke and Gorley, 2015) version 4.7 was used to cluster protein sequences with default settings. This resulted in 612 and 239 protein sequences for the SAT/MET3 and AprBA databases, respectively. These two custom-made databases are also publicly available (see text footnote 1). For the third step of the pathway (reduction of sulfite to sulfide), metagenomes were aligned against a published database of DsrAB (Müller et al., 2014) protein sequences. Alignments were performed using BLAST+ (Camacho et al., 2009) version 2.6.0 (blastp) with an *e* cutoff of 10^−5^, a minimum alignment length of 30 amino acids, and a minimum percent identity of 50%. Positive alignments were normalized to the total pCDS, and the abundance of SAT/MET3, AprBA, and DsrAB sequences was compared between all samples (*n* = 11; PET2 failed normality testing).

### Statistical Analyses

Unless stated otherwise, R (R Core Team, 2017) version 3.4.0 was used for statistical analysis of data. The combination of Shapiro–Wilk tests and quantile-quantile plots were used to test data for normality. One-way ANOVAs were generated using the R package multcomp, using a Tukey’s *post hoc* test with Westfall values.

### Data Availability

All sequence reads were made available through the project accession ERP017130 at the European Nucleotide Archive. The six MAGs were deposited as Madre1, 2, 3, 4, 5, and 6 at GenBank under the accession numbers QZKZ00000000, QZLA00000000, QZLB00000000, QZLC0000000, QZLD00000000, and QZLE00000000, respectively.

## Results

### Microbial Community Composition

**Figure 1:**
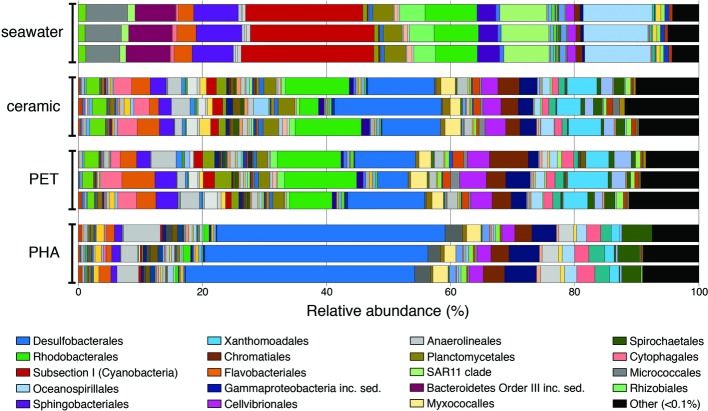
Bar plot showing the relative abundance of microbial orders. Abundances were normalized to the total number of sequences, and orders that constituted to less than 0.1% of the community were grouped under other. The 20 most abundant orders across all samples are displayed in the legend.

Overall trends in the seawater-, ceramic-, PET-, and PHA-associated community compositions were visualized using the taxonomic rank of order based on the normalized proportion of OTUs assigned to each of the 12 samples (three seawater, three ceramic, three PET, and three PHA samples; Figure 1). The comparison of weighted UniFrac values revealed a significant difference between all three biofilm communities (ceramic, PET, and PHA) and the seawater community (PERMANOVA; *p* <0.05). Additionally, a significant difference was found between the PHA biofilm communities and both the ceramic and PET biofilm communities (PERMANOVA; *p* <0.05). By contrast, there was no significant difference between the PET and ceramic biofilm communities. Hierarchical clustering based on a Bray-Curtis resemblance matrix of OTU taxonomic assignments determined that the PET and ceramic biofilm communities were more closely related to the PHA biofilm community than to the seawater community (PERMANOVA; *p* <0.05).

At the phylum level, OTUs belonging to Proteobacteria were the most abundant among all four community types, representing 42, 55, 59, and 72% of all OTUs in seawater, ceramic, PET, and PHA samples, respectively (Supplementary Table S2). Cyanobacteria was the second most abundant phylum in the seawater community (22%), making up a larger proportion of the community compared to the PET and ceramic biofilm communities (~6% for each), and even more so compared to the PHA biofilm community (0.7%). The presence of Chloroflexi (4%), Spirochaetes (4%), and Firmicutes (2%) among the most abundant phyla was unique to the PHA biofilm communities. A detailed report of taxonomic rank based on normalized proportion is provided in Supplementary Table S2.

The OTUs assigned to orders Subsection I of Cyanobacteria, Oceanospirillales, SAR11, and Sphingobacteriales were among the top five most abundant in the seawater community only, making up 20, 11, 8, and 7% of the total OTUs in seawater, respectively. The orders Desulfobacterales, Rhodobacterales, Xanthomondales, and Cellvibrionales were all among the top five most abundant orders in both the PET and ceramic biofilm communities. The abundance of Proteobacteria (72% of all OTUs) within the PHA biofilm community reflected the dominance of the order Desulfobacterales, an order of SRM that represented 37% of all OTUs and more than half of all Proteobacteria. Similar to the trends with phyla, the abundant orders within the PHA biofilm community were unique, with the orders of Gammaproteobacteria *incertae sedis*, Anaerolineales, and Spirochaetales being in the top five most abundantly assigned OTUs in the PHA biofilm communities.

Two genera of Cyanobacteria (*Synechococcus* and *Prochlorococcus*) represented almost 20% of all OTUs within the seawater community but made up a very small proportion (<1%) of all three biofilm communities. Members of uncultured genera from *Desulfobacteraceae, Rhodobacteraceae*, and *Flammeovirgaceae* were among the five most abundant genera in the PET and ceramic biofilm communities, representing almost 20% of all OTUs. Three genera within *Desulfobacteraceae* and an uncultured genus of *Desulfobulbaceae* combined to represent 20% of all OTUs within the PHA biofilm, reinforcing the dominance of SRM in that community (Supplementary Table S2).

### Enrichment of Alpha/Beta Hydrolases

**Figure 2:**
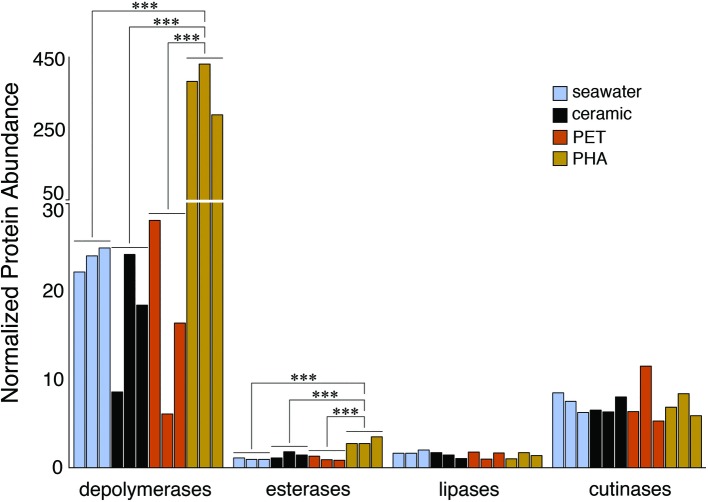
Bar plot showing the relative abundance of depolymerases, esterases, lipases, and cutinases. Gene sequences were normalized to the total number of pCDS in each sample, with individual samples plotted and grouped together based on community association (seawater, ceramic, PET, and PHA). Significance was determined using ANOVA (***p = <0.001, *n* = 3).

**Figure 3:**
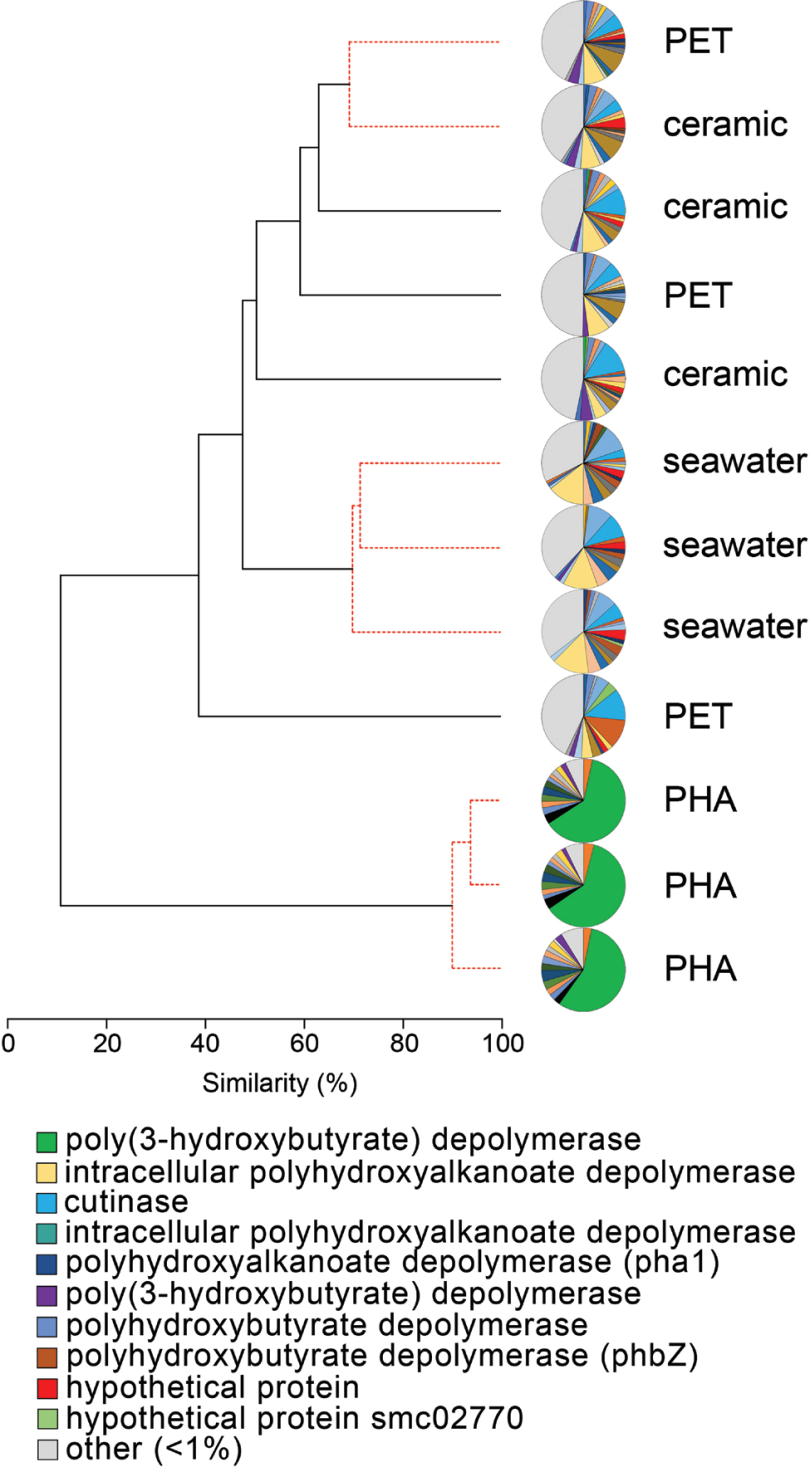
The relatedness of biofilm communities based on a normalized comparison of hydrolase gene pools. The PHB depolymerase, abundant in the PHA communities, is shown in green. Hierarchal clustering was performed based on a Bray-Curtis resemblance matrix. Solid black lines indicate hydrolase gene pools significantly different from one another, while dotted red lines indicate enzyme pools that are not significantly different (SIMPROF analysis; p <0.05). The 10 most abundant enzymes across all samples are displayed in the legend.

The comparison of normalized sequence abundance data revealed a significant enrichment of depolymerase and esterase gene sequences within the PHA biofilm communities versus the seawater, ceramic, and PET biofilm communities (ANOVA; *p* <0.05; Figure 2). The increase in depolymerases was nearly 20-fold, while the increase in esterases was approximately 2.5-fold. The significant increase in depolymerases was largely attributed to a polyhydroxybutyrate (PHB) depolymerase that constituted 60% of all hydrolases in the PHA biofilm communities but less than 0.4% of all hydrolases in other community types (Figure 3). Conversely, no hydrolases were enriched in the PET biofilm community versus either the ceramic biofilm or seawater communities. Hierarchical clustering based on a Bray-Curtis resemblance matrix of hydrolase relative abundance confirmed that PHA biofilm enzyme pools were significantly different from all other community types (SIMPROF; *p* <0.05). Seawater enzyme pools were also unique, while the PET and ceramic biofilms were indistinguishable (Figure 3).

### Relatedness of Polyhydroxybutyrate Depolymerases

To characterize the enriched PHB depolymerases within the PHA biofilm communities, the three PHA metagenomes were co-assembled, resulting in 118,520 contigs totaling 245 Mbp. The N50 of the co-assembly was 2,119 bp, and the lengths of the smallest and longest contigs were 1,000 and 107,130 bp, respectively. The average contig length was 2,251 bp. The PHB depolymerase gene sequence was detected in 46 contigs. The average sequence identity between the 46 PHB depolymerase gene sequences was 54.0% with a range of 7.8–98.9%, and the ML tree illustrated the diversity of those 46 genes along with PHB depolymerase gene sequences from 10 known PHB degraders (Supplementary Figure S3).

### Recovery and Analysis of SRM Genomes

Six high-quality MAGs were recovered from the co-assembled PHA biofilm metagenomes. Initial MAG identification was inferred with CheckM: Madre1 was placed in the genus *Desulfovibrio*, Madre2 in the *Desulfobacteraceae* family, Madre3 in the *Desulfobulbaceae* family, Madre4 in the *Spirochaetaceae* family, and Madre5 and Madre6 within the Gammaproteobacteria order (Table 1). The three SRM MAGs (Madre1, 2, and 3) averaged 391 contigs, a N50 value of 18,335 bp, and a maximum contig length of 76,914 bp. The genome sizes ranged from 3,852,664 to 5,613,049 bp (Madre1 and Madre2, respectively), while the number of genes ranged from 3,829 to 5,179 (Madre1 and Madre2, respectively), and the GC content ranged from 46.9 to 59.8% (Madre3 and Madre1, respectively).

**Table 1:** Six high quality MAGs were recovered from the three PHA co-assembled metagenomes.

MAG	Completeness (%)	Contamination (%)	Quality	Genome size (Mbp)	Contigs	GC%	Taxonomy
Madre1	92.60	1.80	83.60	3.85	434	59.8	*Desulfovibrio*
Madre2	97.40	2.26	86.10	5.61	410	50.5	*Desulfobacteraceae*
Madre3	98.13	2.20	87.13	4.40	330	46.9	*Desulfobulbaceae*
Madre4	54.63	0.00	54.63	1.50	389	50.0	*Spirochaetaceae*
Madre5	76.65	2.26	65.35	2.77	339	39.7	Gammaproteobacteria
Madre6	72.41	1.72	63.81	2.77	201	41.1	Gammaproteobacteria

MAGs with a quality score (% completeness – 5 × % contamination) greater than 50 were considered high quality.

**Figure 4:**
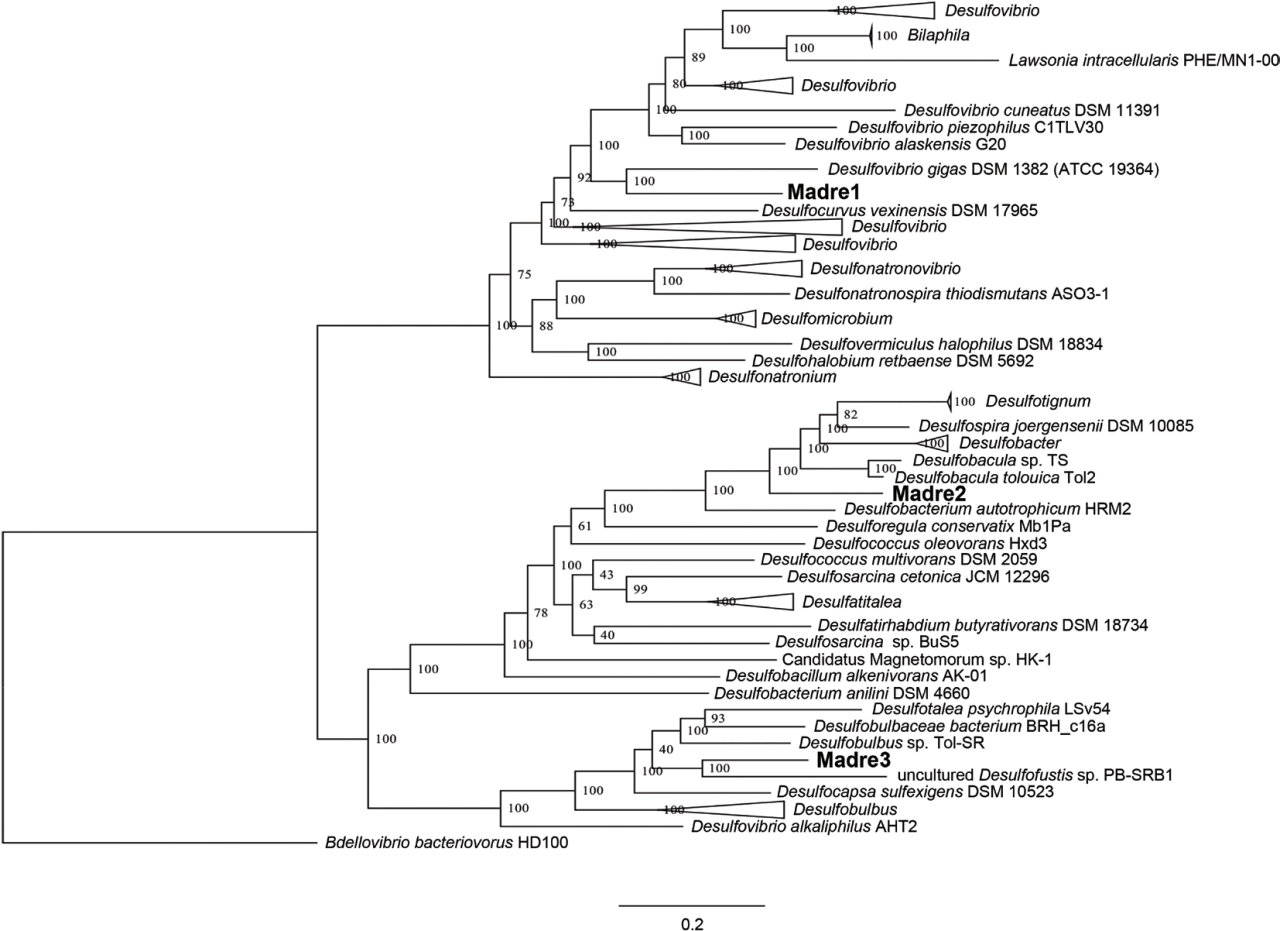
Genome-scale maximum-likelihood tree showing the relatedness of the three PHA biofilm SRM (Madre1, Madre2, and Madre3) to members of the *Desulfovibrionaceae, Desulfobacteraceae*, and *Desulfobulbaceae*. The Madre SRM were highlighted in bold font. Node labels show the bootstrap support values. Branch lengths represent the average number of substitutions per site. The tree was rooted to a more distantly related Deltaproteobacteria that is not a known SRM (*Bdellovibrio bacteriovorans* HD1000).

The identities of the three PHA-associated SRM genomes were further explored by building a genome-scale phylogenetic tree that included 43 *Desulfovibrionaceae*, 21 *Desulfobacteraceae*, and 11 *Desulfobulbaceae* reference genomes (Figure 4). The phylogeny confirmed the initial CheckM-based placement of the three MAGs and further refined their closest relatives. Madre1 was related to the type strain *Desulfovibrio gigas* DSM 1382 (ATCC 19364) (Le Gall, 1963; Morais-Silva et al., 2014). Madre2 was related to the uncultured *Desulfobacula toluolica* Tol2, an aromatic carbon-degrading SRM isolated from a seawater pond in Massachusetts (Wöhlbrand et al., 2013). Madre3 was related to the uncultured *Desulfofustis* sp. PB-SRB1 that was recovered from the “pink berry” consortia of the Sippewissett salt marsh (Wilbanks et al., 2014). However, a species could not be assigned to Madre 1, 2, or 3 given that each showed less than 83% ANI with the 122 *Desulfovibrionaceae*, 107 *Desulfobacteraceae*, and 65 *Desulfobulbaceae* reference genomes available in Genbank. The full tree, without collapsed branches, is included in the supplemental material (Supplementary Figure S4).

Analysis of the three SRM MAGs revealed the presence of SAT/MET3 (Madre1, 2, and 3), AprBA (Madre2, and 3), and DsrAB (Madre1, 2, and 3) protein sequences. Additionally, the alignment of their pCDS to the custom hydrolase database revealed the presence of two distinct PHA depolymerase sequences in both Madre1 and Madre3.

### Dissimilatory Sulfur Reduction Potential

**Figure 5:**
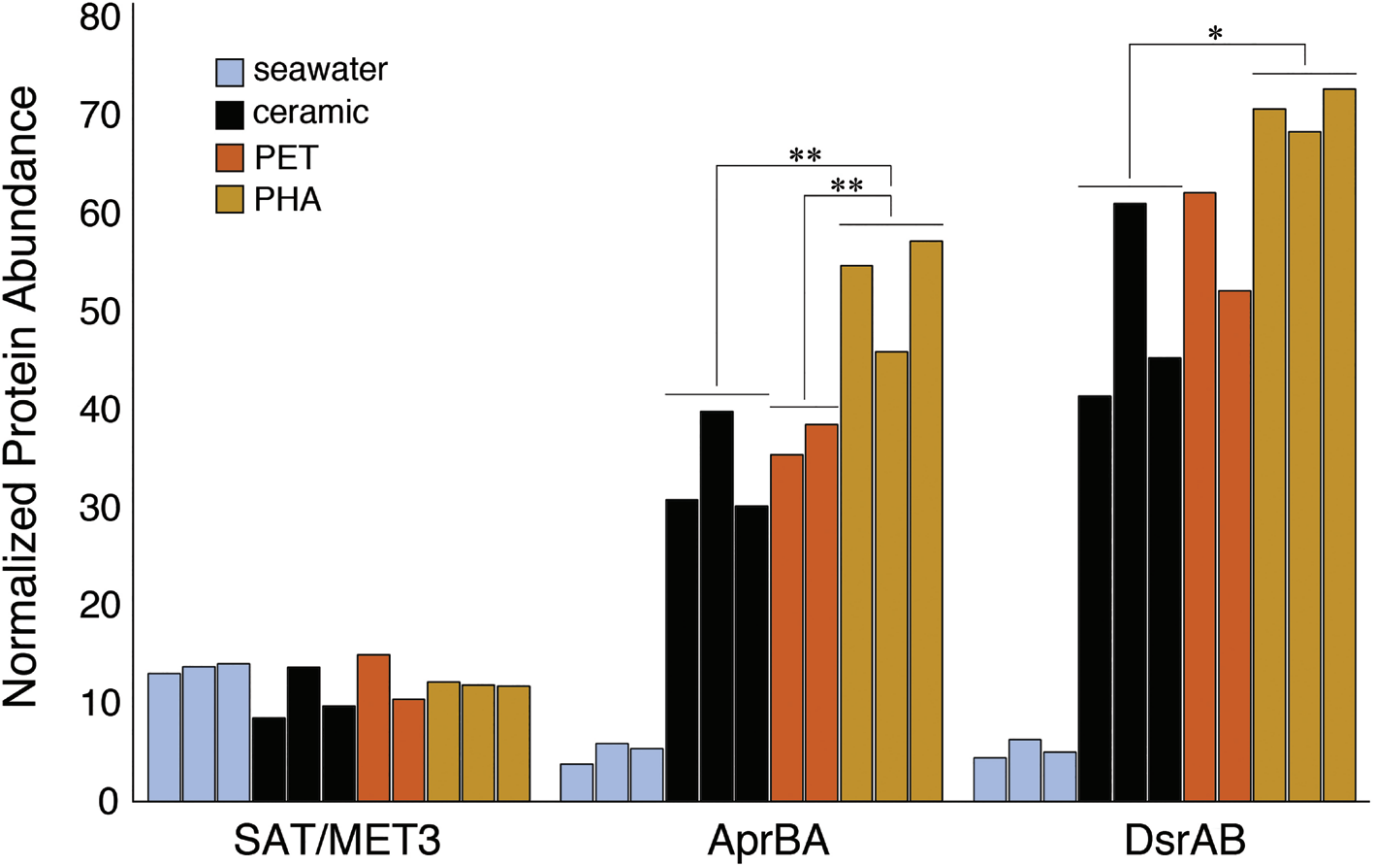
Bar plot showing the relative abundance of sulfate adenylyltransferase (SAT*/*MET3), APS reductase (AprAB), and dissimilatory sulfite reductase (DsrAB) protein sequences. Protein sequences were normalized to the total number of pCDS in each sample, with individual samples plotted and grouped together based on community association (seawater, ceramic, PET, and PHA). Significance was determined using ANOVA (**p <0.01, *p <0.05, *n* = 2–3).

The enrichment of dissimilatory sulfur reduction protein sequences within the PHA biofilm communities was assessed through the alignment of metagenomic pCDS against databases of sulfate adenylyltransferases (SAT/MET3), adenylyl sulfate (APS) reductases (AprBA), and sulfite reductases (DsrAB). The comparison of normalized sequence abundance revealed no significant differences in SAT/MET3 protein abundances. A significant enrichment in AprBA and DsrAB was detected in the PHA biofilm communities (ANOVA; *p* <0.05; Figure 5).

## Discussion

Plastic is a prominent marine pollutant. Numerous studies have characterized microbe-plastic interactions in pelagic systems, yet the effects of plastic pollution on benthic microbiota and biogeochemical cycling remain unclear. To address this knowledge gap, we conducted an *in situ* microcosm designed to ask how the benthic microbial community responds to plastic and bioplastic in coastal marine sediments. Coastal sediments are especially vulnerable to plastic loading due to their proximity to population centers and urban environments (Barnes et al., 2009), and the majority of plastic debris entering the oceans accumulates in coastal zones (Barnes et al., 2009; Thompson et al., 2009).

The comparison between surface-associated and free-living lifestyles has been a long-studied question in microbial ecology and, thereby, considerable literature is available to inform the study of surface-associated bacterial communities (Biddanda and Pomeroy, 1988; Karner and Herndl, 1992; DeLong et al., 1993; Crump et al., 1999; Hollibaugh et al., 2000; Kellogg and Deming, 2014). Based on this literature, it was hypothesized that all surface-associated communities (i.e., ceramic, plastic, and bioplastic) would be distinct in comparison to free-living communities, and the results of this study supported that hypothesis. The more salient question was the distinction between different surfaces, and this study revealed that PET was not colonized by a distinct community in comparison to the ceramic biofilm control. This finding is supported by a recent study demonstrating that pelagic microbial communities associated with PET were indistinguishable from those associated with a glass biofilm control (Oberbeckmann et al., 2016). Similarly, it was previously demonstrated that inert surfaces such as glass, ceramic, and coral skeleton have little influence on marine microbial community composition (Witt et al., 2011). The lack of distinction between PET and ceramic could also be due to the remote location of the study site seeing that previous reports of plastic degradation have occurred in highly polluted habitats that would select for and enrich plastic degraders (Nanda et al., 2010; Singh and Gupta, 2014; Yoshida et al., 2016).

Whereas PET was not colonized by a distinct microbial community, the introduction of PHA promoted a significant and distinct response. In particular, SRM were the dominant members of the PHA-associated assemblage (see Figure 3). Typically, the three most common SRM families (*Desulfobacteraceae, Desulfobulbaceae,* and *Desulfovibrionaceae*) account for between 5 and 20% of the total bacterial community in estuarine sediments (Bowen et al., 2012; Cheung et al., 2018). In this study, these three families made up a similar fraction of the plastic- and ceramic-associated benthic communities (9 and 12%, respectively) but they accounted for over 39% of the PHA-associated benthic community.

Naturally occurring PHAs are carbon and energy storage polymers that are accumulated as intracellular granules that aid survival during periods of nutrient limitation and environmental stress (Dawes and Senior, 1973; Obruca et al., 2018). The ability to produce and degrade PHA is thought to be widespread (Reddy et al., 2003) but its prevalence among SRM and its importance in carbon and sulfur cycling has not been thoroughly characterized. A previous study has reported PHA accumulation in *Desulfococcus multivorans, Desulfobotulus sapovorans, Desulfonema magnum*, and *Desulfobacterium autotrophicum* (Hai et al., 2004). Importantly, two culture-dependent studies have shown that addition of PHA to incubation bottles was correlated with sulfide production in anoxic lake sediments (Mas-Castellà et al., 1995; Urmeneta et al., 1995). Taken together, the accumulation of PHA in SRM, the PHA stimulation of sulfide production, and the PHA-selection of SRM (in this study) indicate that PHA is an important carbon source for sulfate reduction. However, it remains unknown if the sulfate reducers in our study can directly degrade PHA or if they rely on primary fermenters to first degrade the polymer into simple organic substrates.

**Figure 6:**
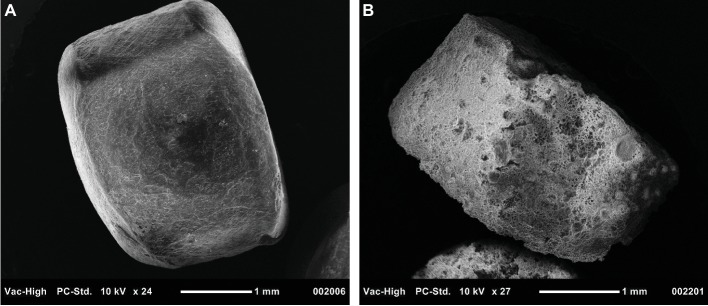
Scanning electron microscopy image of PHA pellets used to visualize signs of biodegradation. Panel **(A)** displays a pellet that was not exposed to benthic microbial communities, while Panel **(B)** shows a pellet exposed for 230 days.

A key reaction in PHA degradation is its depolymerization. In this study, a nearly 20-fold increase in the abundance of depolymerases indicated that PHA stimulated the growth of PHA-degrading bacteria. Further, the large diversity of the 46 depolymerases, recovered from the metagenome-assembled genomes, suggested these enzymes were distributed across a mixed microbial consortium. Although we did not measure enzyme activity, the increased abundance and diversity of these depolymerases support the hypothesis that PHA biofilms were sites of enhanced enzyme activity. Additional support for this hypothesis will be made available in a parallel and forthcoming 15-month study wherein weight loss and scanning electron microscopy were used to quantify PHA degradation at the same study site (Figure 6).

Previous studies have shown, using adenylyl sulfate reductases (*aprBA*) and dissimilatory sulfite reductases (*dsrAB*) as functional markers, that sulfate reduction is more diverse and widespread than previously thought (Müller et al., 2014; Anantharaman et al., 2018; Hausmann et al., 2018). Further, the detection of *apr* and *dsr* genes in new phyla, made possible through the construction of genomes from metagenomes, has expanded the known diversity of sulfate-reducing microorganisms (Anantharaman et al., 2018; Hausmann et al., 2018). Here, we demonstrated that bioplastic communities were significantly enriched with both sulfate and sulfite reductases. We also reconstructed genomes from metagenomes to discover new *Desulfovibrio, Desulfobacula*, and *Desulfofustis* species (Madre 1, 2, and 3, respectively). While it remains unknown if SRM possessing depolymerases (i.e., Madre1 and Madre 3) can directly degrade PHA, an ongoing study will shed light on their growth requirements. Despite this open question, the increased abundance of sulfate-reducing enzymes and the discovery of three uncultured sulfate-reducing species lend additional support to the hypothesis that sulfate reduction in marine sediments is stimulated by the addition of PHA.

In a scenario where bioplastic use and pollution are more common, what remains unclear is the larger effect of the PHA-selection for SRM. Sulfate-reducing bacteria play a critical role in biogeochemical cycling as sulfate reduction is responsible for ~50% of organic carbon mineralization in marine sediments (Jørgensen, 1982), and the stimulation of SRM has been shown to suppress the growth of methanogenic archaea through the diversion of carbon flow from methane to carbon dioxide (Gauci et al., 2004). For example, the stimulation of SRM in coastal rice fields, through the application of sulfate-containing amendments, was shown to inhibit methane production (Lindau et al., 1994; Denier van der Gon et al., 2001). It is thus possible that bioplastic pollution and its stimulation of SRM could have unintended consequences that affect the balance between sulfate reduction and methanogenesis. More generally, it is also possible that bioplastic loading could alter the natural syntrophic cycling of PHA in marine sediments.

In conclusion, this study demonstrated that the introduction of plastic had no measurable effect on the benthic microbial community. By contrast, the introduction of bioplastic selected for a distinct microbial community that was enriched for hydrolases and dominated by SRM. Recovered genomes, representing the three most common families of SRM, contained depolymerases as well as *aprBA* and *dsrAB* reductases. Findings indicate that SRM play an important role in PHA degradation in coastal marine sediments. Given that sulfate reduction is a key process in the oceanic sulfur cycle, we recommend that future scientific investigation, government legislation, and best-management practices related to plastic pollution consider the effects of plastic as well as bio-based alternatives on benthic biogeochemical activities.

## Data Availability

The datasets generated for this study can be found in ENA/EBI Metagenomics & NCBI, ERP017130 & QZKZ00000000, QZLA00000000, QZLB00000000, QZLC0000000, QZLD00000000, QZLE00000000.

## Author Contributions

All authors contributed to this study’s design, implementation, analyses, and writing.

### Conflict of Interest Statement

The authors declare that the research was conducted in the absence of any commercial or financial relationships that could be construed as a potential conflict of interest.

## Footnotes

^1^turnerlab.tamucc.edu/research/databases

^2^http://tree.bio.ed.ac.uk/software/figtree/

## Supplementary Material

TABLE S1 Library preparation and sequencing information.

TABLE S2 The top five members from each taxonomic rank based on normalized proportion per sample. Means (*n* = 3) plus or minus the standard error of mean are reported for each of the taxonomic assignments.

FIGURE S1 Map of sampling location showing the Upper Laguna Madre (ULM) along the northwestern Gulf of Mexico (Texas, USA) with the sampling site labeled.

FIGURE S2 Example of the sampling device designed for this experiment. Each sampling device **(D)** consisted of a PVC frame containing four MicroCaps: **(A)** polyethylene terephthalate (PET), **(B)** polyhydroxyalkanoate (PHA), or **(C)** ceramic pellets. A total of twelve MicroCaps (*n* = 4 of each sample type) were deployed at the water-sediment interface using a randomized experimental design as shown in panel **(D)**.

FIGURE S3 Maximum-likelihood tree showing the diversity of the 46 enriched PHB depolymerase gene sequences. Node labels show the bootstrap support values. Branch lengths represent the average number of substitutions per site. The tree was rooted to the original PHB depolymerase sequence.

FIGURE S4 The complete phylogenetic tree containing the three PHA biofilm SRBs recovered in this study (highlighted in yellow). The coloration of labels corresponds to different taxa contained within the tree. Branch labels show the bootstrap support values. Unlabeled nodes have support values of 100. Branch lengths represent the average number of substitutions per site. The tree is rooted to a distantly related Deltaproteobacteria (*Bdellovibrio bacteriovorans*).

## References

[ref1] Aeschelmann F. Carus M. (2015). Biobased building blocks and polymers in the world: capacities, production, and applications – status quo and trends towards 2020. Ind. Biotechnol. 11, 154–159. 10.1089/ind.2015.28999.fae

[ref2] Anantharaman K. Hausmann B. Jungbluth S. P. Kantor R. S. Lavy A. Warren L. A.. (2018). Expanded diversity of microbial groups that shape the dissimilatory sulfur cycle. ISME J. 12, 1715–1728. 10.1038/s41396-018-0078-0, PMID: 29467397 PMC6018805

[ref3] Andrady A. L. (2011). Microplastics in the marine environment. Mar. Pollut. Bull. 62, 1596–1605. 10.1016/j.marpolbul.2011.05.030, PMID: 21742351

[ref4] Azam F. Long R. A. (2001). Oceanography: sea snow microcosms. Nature 414, 495–498. 10.1038/35107174, PMID: 11734832

[ref5] Azam F. Smith D. C. Steward G. F. Hagström Å. (1994). Bacteria-organic matter coupling and its significance for oceanic carbon cycling. Microb. Ecol. 28, 167–179. 10.1007/BF00166806, PMID: 24186443

[ref6] Barnes D. K. Galgani F. Thompson R. C. Barlaz M. (2009). Accumulation and fragmentation of plastic debris in global environments. Philos. Trans. R. Soc. Lond. Ser. B Biol. Sci. 364, 1985–1998. 10.1098/rstb.2008.0205 19528051 PMC2873009

[ref7] Biddanda B. A. Pomeroy L. R. (1988). Microbial aggregation and degradation of phytoplankton-derived detritus in seawater. I. Microbial succession. Mar. Ecol. Prog. Ser. 42, 79–88. 10.3354/meps042079

[ref8] Bolger A. M. Lohse M. Usadel B. (2014). Trimmomatic: a flexible trimmer for Illumina sequence data. Bioinformatics 30, 2114–2120. 10.1093/bioinformatics/btu170, PMID: 24695404 PMC4103590

[ref9] Bowen J. L. Morrison H. G. Hobbie J. E. Sogin M. L. (2012). Salt marsh sediment diversity: a test of the variability of the rare biosphere among environmental replicates. ISME J. 6, 2014–2023. 10.1038/ismej.2012.47, PMID: 22739491 PMC3475368

[ref10] Brettin T. Davis J. J. Disz T. Edwards R. A. Gerdes S. Olsen G. J. (2015). RASTtk: a modular and extensible implementation of the RAST algorithm for building custom annotation pipelines and annotating batches of genomes. Sci. Rep. 5:8365. 10.1038/srep08365 25666585 PMC4322359

[ref11] Bryant J. A. Clemente T. M. Viviani D. A. Fong A. A. Thomas K. A. Kemp P. (2016). Diversity and activity of communities inhabiting plastic debris in the North Pacific Gyre. mSystems 1, e00024–e00016. 10.1128/mSystems.00024-16 27822538 PMC5069773

[ref12] Camacho C. Coulouris G. Avagyan V. Ma N. Papadopoulos J. Bealer K. (2009). BLAST+: architecture and applications. BMC Bioinformatics 10:421. 10.1186/1471-2105-10-421 20003500 PMC2803857

[ref13] Capella-Gutiérrez S. Silla-Martínez J. M. Gabaldón T. (2009). trimAl: a tool for automated alignment trimming in large-scale phylogenetic analyses. Bioinformatics 25, 1972–1973. 10.1093/bioinformatics/btp348, PMID: 19505945 PMC2712344

[ref14] Caporaso J. G. Kuczynski J. Stombaugh J. Bittinger K. Bushman F. D. Costello E. K.. (2010). QIIME allows analysis of high-throughput community sequencing data. Nat. Methods 7, 335–336. 10.1038/nmeth.f.303, PMID: 20383131 PMC3156573

[ref15] Carson H. S. Nerheim M. S. Carroll K. A. Eriksen M. (2013). The plastic-associated microorganisms of the North Pacific Gyre. Mar. Pollut. Bull. 75, 126–132. 10.1016/j.marpolbul.2013.07.054, PMID: 23993070

[ref16] Cheung M. K. Wong C. K. Chu K. H. Kwan H. S. (2018). Community structure, dynamics and interactions of bacteria, Archaea and fungi in subtropical coastal wetland sediments. Sci. Rep. 8:14397. 10.1038/s41598-018-32529-5, PMID: 30258074 PMC6158284

[ref17] Clarke K. Gorley R. (2015). PRIMER v7: User manual/tutorial. (Plymouth, UK: PRIMER-E Ltd).

[ref18] Clarke K. R. Somerfield P. J. Gorley R. N. (2008). Testing of null hypotheses in exploratory community analyses: similarity profiles and biota-environment linkage. J. Exp. Mar. Biol. Ecol. 366, 56–69. 10.1016/j.jembe.2008.07.009

[ref19] Cock P. J. A. Antao T. Chang J. T. Chapman B. A. Cox C. J. Dalke A.. (2009). Biopython: freely available python tools for computational molecular biology and bioinformatics. Bioinformatics 25, 1422–1423. 10.1093/bioinformatics/btp163, PMID: 19304878 PMC2682512

[ref20] Crump B. C. Armbrust E. V. Baross J. A. (1999). Phylogenetic analysis of particle-attached and free-living bacterial communities in the Columbia River, its estuary, and the adjacent coastal ocean. Appl. Environ. Microbiol. 65, 3192–3204. PMID: 10388721 10.1128/aem.65.7.3192-3204.1999 PMC91474

[ref21] Dang H. Lovell C. R. (2016). Microbial surface colonization and biofilm development in marine environments. Microbiol. Mol. Biol. Rev. 80, 91–138. 10.1128/MMBR.00037-15 26700108 PMC4711185

[ref22] Dawes E. A. Senior P. J. (1973). “The role and regulation of energy reserve polymers in micro-organisms” in Advances in Microbial Physiology. eds. Rose A. H. Tempest D. W. (London, UK: Academic Press), 135–266. 10.1016/s0065-2911(08)60088-0 4594739

[ref23] De Tender C. A. Devriese L. I. Haegeman A. Maes S. Ruttink T. Dawyndt P. (2015). Bacterial community profiling of plastic litter in the Belgian part of the North Sea. Environ. Sci. Technol. 49, 9629–9638. 10.1021/acs.est.5b01093, PMID: 26204244

[ref24] DeLong E. F. Franks D. G. Alldredge A. L. (1993). Phylogenetic diversity of aggregate-attached vs. free-living marine bacterial assemblages. Limnol. Oceanogr. 38, 924–934. 10.4319/lo.1993.38.5.0924

[ref25] Denier van der Gon H. A. van Bodegom P. M. Wassmann R. Lantin R. S. Metra-Corton T. M. (2001). Sulfate-containing amendments to reduce methane emissions from rice fields: mechanisms, effectiveness and costs. Mitig. Adapt. Strat. Gl. 6, 71–89. 10.1023/A:1011380916490

[ref26] Edgar R. C. (2004). MUSCLE: multiple sequence alignment with high accuracy and high throughput. Nucleic Acids Res. 32, 1792–1797. 10.1093/nar/gkh340, PMID: 15034147 PMC390337

[ref27] Eich A. Mildenberger T. Laforsch C. Weber M. (2015). Biofilm and diatom succession on polyethylene (PE) and biodegradable plastic bags in two marine habitats: early signs of degradation in the pelagic and benthic zone? PLoS One 10:e0137201. 10.1371/journal.pone.0137201, PMID: 26394047 PMC4578875

[ref28] Enright A. J. Van Dongen S. Ouzounis C. A. (2002). An efficient algorithm for large-scale detection of protein families. Nucleic Acids Res. 30, 1575–1584. 10.1093/nar/30.7.1575, PMID: 11917018 PMC101833

[ref29] European Commission (2018). Directive of the European parliament and of the council on the reduction of the impact of certain plastic products on the environment. (Brussels, Belgium: European Commission).

[ref30] Gauci V. Matthews E. Dise N. Walter B. Koch D. Granberg G. (2004). Sulfur pollution suppression of the wetland methane source in the 20th and 21st centuries. Proc. Natl. Acad. Sci. USA 101, 12583–12587. 10.1073/pnas.0404412101 15297612 PMC515100

[ref31] Geyer R. Jambeck J. R. Law K. L. (2017). Production, use, and fate of all plastics ever made. Sci. Adv. 3:e1700782. 10.1126/sciadv.1700782, PMID: 28776036 PMC5517107

[ref32] Hai T. Lange D. Rabus R. Steinbuchel A. (2004). Polyhydroxyalkanoate (PHA) accumulation in sulfate-reducing bacteria and identification of a class III PHA synthase (PhaEC) in *Desulfococcus multivorans*. Appl. Environ. Microbiol. 70, 4440–4448. 10.1128/AEM.70.8.4440-4448.2004, PMID: 15294771 PMC492432

[ref33] Harke M. J. Davis T. W. Watson S. B. Gobler C. J. (2015). Nutrient-controlled niche differentiation of western Lake Erie cyanobacterial populations revealed via metatranscriptomic surveys. Environ. Sci. Technol. 50, 604–615. 10.1021/acs.est.5b03931 26654276

[ref34] Harrison J. P. Schratzberger M. Sapp M. Osborn A. M. (2014). Rapid bacterial colonization of low-density polyethylene microplastics in coastal sediment microcosms. BMC Microbiol. 14, 1–15. 10.1186/s12866-014-0232-4 PMC4177575 25245856

[ref35] Hausmann B. Pelikan C. Herbold C. W. Köstlbacher S. Albertsen M. Eichorst S. A.. (2018). Peatland Acidobacteria with a dissimilatory sulfur metabolism. ISME J. 12, 1729–1742. 10.1038/s41396-018-0077-1, PMID: 29476143 PMC6018796

[ref36] Hollibaugh J. T. Wong P. S. Murrell M. C. (2000). Similarity of particle-associated and free-living bacterial communities in northern San Francisco Bay, California. Aquat. Microb. Ecol. 21, 103–114. 10.3354/ame021103

[ref37] Hotelier T. Renault L. Cousin X. Negre V. Marchot P. Chatonnet A. (2004). ESTHER, the database of the α/β-hydrolase fold superfamily of proteins. Nucleic Acids Res. 32, D145–D147. 10.1093/nar/gkh141 14681380 PMC308875

[ref38] Hyatt D. Chen G.-L. Locascio P. F. Land M. L. Larimer F. W. Hauser L. J. (2010). Prodigal: prokaryotic gene recognition and translation initiation site identification. BMC Bioinformatics 11:119. 10.1186/1471-2105-11-119 20211023 PMC2848648

[ref39] Jain C. Rodriguez-R L. M. Phillippy A. M. Konstantinidis K. T. Aluru S. (2018). High-throughput ANI analysis of 90K prokaryotic genomes reveals clear species boundaries. Nat. Commun. 9:5114. 10.1038/s41467-018-07641-9 30504855 PMC6269478

[ref40] Jambeck J. R. Geyer R. Wilcox C. Siegler T. R. Perryman M. Andrady A.. (2015). Plastic waste inputs from land into the ocean. Science 347, 768–771. 10.1126/science.1260352, PMID: 25678662

[ref41] John J. S. (2011). SeqPrep. Available at: https://github.com/jstjohn/SeqPrep (Accessed September 14, 2016).

[ref42] Jørgensen B. B. (1977). The sulfur cycle of a coastal marine sediment (Limfjorden, Denmark). Limnol. Oceanogr. 22, 814–832. 10.4319/lo.1977.22.5.0814

[ref43] Jørgensen B. B. (1982). Mineralization of organic matter in the sea bed—the role of sulphate reduction. Nature 296, 643–645. 10.1038/296643a0

[ref44] Kalyaanamoorthy S. Minh B. Q. Wong T. K. F. von Haeseler A. Jermiin L. S. (2017). ModelFinder: fast model selection for accurate phylogenetic estimates. Nat. Methods 14, 587–589. 10.1038/nmeth.4285, PMID: 28481363 PMC5453245

[ref45] Kang D. D. Froula J. Egan R. Wang Z. (2015). MetaBAT, an efficient tool for accurately reconstructing single genomes from complex microbial communities. PeerJ 3:e1165. 10.7717/peerj.1165, PMID: 26336640 PMC4556158

[ref46] Karner M. Herndl G. J. (1992). Extracellular enzymatic activity and secondary production in free-living and marine-snow-associated bacteria. Mar. Biol. 113, 341–347.

[ref47] Kellogg C. T. E. Deming J. W. (2014). Particle-associated extracellular enzyme activity and bacterial community composition across the Canadian Arctic Ocean. FEMS Microbiol. Ecol. 89, 360–375. 10.1111/1574-6941.12330, PMID: 24666253

[ref48] Klump J. V. Martens C. S. (1981). Biogeochemical cycling in an organic rich coastal marine basin—II. Nutrient sediment-water exchange processes. Geochim. Cosmochim. Acta 45, 101–121. 10.1016/0016-7037(81)90267-2

[ref49] Le Gall J. (1963). A new species of *Desulfovibrio*. J. Bacteriol. 86, 1120–1120. 14080783 10.1128/jb.86.5.1120-1120.1963 PMC278577

[ref50] Lee J. Yi H. Chun J. (2011). rRNASelector: a computer program for selecting ribosomal RNA encoding sequences from metagenomic and metatranscriptomic shotgun libraries. J. Microbiol. 49, 689–691. 10.1007/s12275-011-1213-z, PMID: 21887657

[ref51] Li H. Durbin R. (2010). Fast and accurate long-read alignment with burrows-wheeler transform. Bioinformatics 26, 589–595. 10.1093/bioinformatics/btp698, PMID: 20080505 PMC2828108

[ref52] Li D. Liu C.-M. Luo R. Sadakane K. Lam T.-W. (2015). MEGAHIT: an ultra-fast single-node solution for large and complex metagenomics assembly via succinct de Bruijn graph. Bioinformatics 31, 1674–1676. 10.1093/bioinformatics/btv033, PMID: 25609793

[ref53] Lindau C. W. Alford D. P. Bollich P. K. Linscombe S. D. (1994). Inhibition of methane evolution by calcium sulfate addition to flooded rice. Plant Soil 158, 299–301. 10.1007/BF00009503

[ref54] Lozupone C. Lladser M. E. Knights D. Stombaugh J. Knight R. (2011). UniFrac: an effective distance metric for microbial community comparison. ISME J. 5, 169–172. 10.1038/ismej.2010.133, PMID: 20827291 PMC3105689

[ref55] Mas-Castellà J. Urmeneta J. Lafuente R. Navarrete A. Guerrero R. (1995). Biodegradation of poly-β-hydroxyalkanoates in anaerobic sediments. Int. Biodeterior. Biodegrad. 35, 155–174. 10.1016/0964-8305(95)00066-E

[ref56] Minh B. Q. Nguyen M. A. T. von Haeseler A. (2013). Ultrafast approximation for phylogenetic bootstrap. Mol. Biol. Evol. 30, 1188–1195. 10.1093/molbev/mst024, PMID: 23418397 PMC3670741

[ref57] Mitchell A. Bucchini F. Cochrane G. Denise H. Hoopen P.t. Fraser M.. (2016). EBI metagenomics in 2016 - an expanding and evolving resource for the analysis and archiving of metagenomic data. Nucleic Acids Res. 44, D595–D603. 10.1093/nar/gkv1195, PMID: 26582919 PMC4702853

[ref58] Morais-Silva F. O. Rezende A. M. Pimentel C. Santos C. I. Clemente C. Varela–Raposo A.. (2014). Genome sequence of the model sulfate reducer *Desulfovibrio gigas*: a comparative analysis within the *Desulfovibrio* genus. Microbiology 3, 513–530. 10.1002/mbo3.184, PMID: 25055974 PMC4287179

[ref59] Müller A. L. Kjeldsen K. U. Rattei T. Pester M. Loy A. (2014). Phylogenetic and environmental diversity of DsrAB-type dissimilatory (bi)sulfite reductases. ISME J. 9, 1152–1165. 10.1038/ismej.2014.208 25343514 PMC4351914

[ref60] Nanda S. Sahu S. Abraham J. (2010). Studies on the biodegradation of natural and synthetic polyethylene by *Pseudomonas* spp. J. Appl. Sci. Environ. Manag. 14, 57–60. 10.4314/jasem.v14i2.57839

[ref61] Nauendorf A. Krause S. Bigalke N. K. Gorb E. V. Gorb S. N. Haeckel M.. (2016). Microbial colonization and degradation of polyethylene and biodegradable plastic bags in temperate fine-grained organic-rich marine sediments. Mar. Pollut. Bull. 103, 168–178. 10.1016/j.marpolbul.2015.12.024, PMID: 26790603

[ref62] Nguyen L.-T. Schmidt H. A. von Haeseler A. Minh B. Q. (2015). IQ-TREE: a fast and effective stochastic algorithm for estimating maximum-likelihood phylogenies. Mol. Biol. Evol. 32, 268–274. 10.1093/molbev/msu300, PMID: 25371430 PMC4271533

[ref63] Notredame C. Higgins D. G. Heringa J. (2000). T-coffee: a novel method for fast and accurate multiple sequence alignment. J. Mol. Biol. 302, 205–217. 10.1006/jmbi.2000.4042, PMID: 10964570

[ref64] Oberbeckmann S. Loeder M. G. Gerdts G. Osborn A. M. (2014). Spatial and seasonal variation in diversity and structure of microbial biofilms on marine plastics in northern European waters. FEMS Microbiol. Ecol. 90, 478–492. 10.1111/1574-6941.12409, PMID: 25109340

[ref65] Oberbeckmann S. Osborn A. M. Duhaime M. B. (2016). Microbes on a bottle: substrate, season and geography influence community composition of microbes colonizing marine plastic debris. PLoS One 11:e0159289. 10.1371/journal.pone.0159289, PMID: 27487037 PMC4972250

[ref66] Obruca S. Sedlacek P. Koller M. Kucera D. Pernicova I. (2018). Involvement of polyhydroxyalkanoates in stress resistance of microbial cells: biotechnological consequences and applications. Biotechnol. Adv. 36, 856–870. 10.1016/j.biotechadv.2017.12.006, PMID: 29248684

[ref67] Ondov B. D. Treangen T. J. Melsted P. Mallonee A. B. Bergman N. H. Koren S. (2016). Mash: fast genome and metagenome distance estimation using MinHash. Genome Biol. 17:132. 10.1186/s13059-016-0997-x 27323842 PMC4915045

[ref68] Ortiz-Álvarez R. Fierer N. de los Ríos A. Casamayor E. O. Barberán A. (2018). Consistent changes in the taxonomic structure and functional attributes of bacterial communities during primary succession. ISME J. 12, 1658–1667. 10.1038/s41396-018-0076-2, PMID: 29463893 PMC6018800

[ref69] Parks D. H. Imelfort M. Skennerton C. T. Hugenholtz P. Tyson G. W. (2015). CheckM: assessing the quality of microbial genomes recovered from isolates, single cells, and metagenomes. Genome Res. 25, 1043–1055. 10.1101/gr.186072.114, PMID: 25977477 PMC4484387

[ref70] Parks D. H. Rinke C. Chuvochina M. Chaumeil P.-A. Woodcroft B. J. Evans P. N.. (2017). Recovery of nearly 8,000 metagenome-assembled genomes substantially expands the tree of life. Nat. Microbiol. 2, 1533–1542. 10.1038/s41564-017-0012-7, PMID: 28894102

[ref71] Pauli N.-C. Petermann J. S. Lott C. Weber M. (2017). Macrofouling communities and the degradation of plastic bags in the sea: an *in situ* experiment. R. Soc. Open Sci. 4:e170549. 10.1098/rsos.170549, PMID: 29134070 PMC5666253

[ref72] Ploug H. Grossart H.-P. Azam F. Jørgensen B. B. (1999). Photosynthesis, respiration, and carbon turnover in sinking marine snow from surface waters of Southern California bight: implications for the carbon cycle in the ocean. Mar. Ecol. Prog. Ser. 179, 1–11. 10.3354/meps179001

[ref73] Quast C. Pruesse E. Yilmaz P. Gerken J. Schweer T. Yarza P.. (2013). The SILVA ribosomal RNA gene database project: improved data processing and web-based tools. Nucleic Acids Res. 41, D590–D596. 10.1093/nar/gks1219, PMID: 23193283 PMC3531112

[ref74] R Core Team (2017). R: A language and environment for statistical computing. (Vienna, Austria: R Foundation for Statistical Computing). Available at: https://www.R-project.org/

[ref75] Reddy C. S. K. Ghai R. Rashmi Kalia V. C. (2003). Polyhydroxyalkanoates: an overview. Bioresour. Technol. 87, 137–146. 10.1016/S0960-8524(02)00212-2, PMID: 12765352

[ref76] Rho M. Tang H. Ye Y. (2010). FragGeneScan: predicting genes in short and error-prone reads. Nucleic Acids Res. 38:e191. 10.1093/nar/gkq747, PMID: 20805240 PMC2978382

[ref77] Seymour J. R. Amin S. A. Raina J.-B. Stocker R. (2017). Zooming in on the phycosphere: the ecological interface for phytoplankton–bacteria relationships. Nat. Microbiol. 2:17065. 10.1038/nmicrobiol.2017.65, PMID: 28555622

[ref78] Singh J. Gupta K. (2014). Screening and identification of low density polyethylene (LDPE) degrading soil fungi isolated from polythene polluted sites around Gwalior City (MP). Int. J. Curr. Microbiol. App. Sci. 3, 443–448.

[ref79] Skennerton C. T. Haroon M. F. Briegel A. Shi J. Jensen G. J. Tyson G. W.. (2016). Phylogenomic analysis of Candidatus ‘Izimaplasma’species: free-living representatives from a Tenericutes clade found in methane seeps. ISME J. 10, 2679–2692. 10.1038/ismej.2016.55, PMID: 27058507 PMC5113845

[ref80] Stamatakis A. (2014). RAxML version 8: a tool for phylogenetic analysis and post-analysis of large phylogenies. Bioinformatics 30, 1312–1313. 10.1093/bioinformatics/btu033, PMID: 24451623 PMC3998144

[ref81] Talavera G. Castresana J. (2007). Improvement of phylogenies after removing divergent and ambiguously aligned blocks from protein sequence alignments. Syst. Biol. 56, 564–577. 10.1080/10635150701472164, PMID: 17654362

[ref82] Thompson R. C. Moore C. J. vom Saal F. S. Swan S. H. (2009). Plastics, the environment and human health: current consensus and future trends. Philos. Trans. R. Soc. Lond. Ser. B Biol. Sci. 364, 2153–2166. 10.1098/rstb.2009.0053 19528062 PMC2873021

[ref83] Thompson R. C. Olsen Y. Mitchell R. P. Davis A. Rowland S. J. John A. W. G.. (2004). Lost at sea: where is all the plastic? Science 304:838. 10.1126/science.1094559, PMID: 15131299

[ref84] Tunnell J. W. (2002). “Geography, climate, and hydrography” in The Laguna Madre of Texas and Tamaulipas. eds. Tunnell J. W. Judd F. W. (College Station, Texas: Texas A&M University Press), 7–27.

[ref85] UNEP (2018). Single-use plastics: A roadmap for sustainability. (Nairobi, Kenya: United Nations Environment Programme).

[ref86] Urmeneta J. Mas-Castella J. Guerrero R. (1995). Biodegradation of poly-(beta)-hydroxyalkanoates in a lake sediment sample increases bacterial sulfate reduction. Appl. Environ. Microbiol. 61, 2046–2048. PMID: 16535034 10.1128/aem.61.5.2046-2048.1995 PMC1388452

[ref87] Van Cauwenberghe L. Devriese L. Galgani F. Robbens J. Janssen C. R. (2015). Microplastics in sediments: a review of techniques, occurrence and effects. Mar. Environ. Res. 111, 5–17. 10.1016/j.marenvres.2015.06.007, PMID: 26095706

[ref88] Vigneron A. Cruaud P. Alsop E. de Rezende J. R. Head I. M. Tsesmetzis N. (2018). Beyond the tip of the iceberg; a new view of the diversity of sulfite- and sulfate-reducing microorganisms. ISME J. 12, 2096–2099. 10.1038/s41396-018-0155-4, PMID: 29805176 PMC6052056

[ref89] Wattam A. R. Davis J. J. Assaf R. Boisvert S. Brettin T. Bun C.. (2017). Improvements to PATRIC, the all-bacterial bioinformatics database and analysis resource center. Nucleic Acids Res. 45, D535–D542. 10.1093/nar/gkw1017, PMID: 27899627 PMC5210524

[ref90] Wilbanks E. G. Jaekel U. Salman V. Humphrey P. T. Eisen J. A. Facciotti M. T.. (2014). Microscale sulfur cycling in the phototrophic pink berry consortia of the Sippewissett salt marsh. Environ. Microbiol. 16, 3398–3415. 10.1111/1462-2920.12388, PMID: 24428801 PMC4262008

[ref91] Witt V. Wild C. Uthicke S. (2011). Effect of substrate type on bacterial community composition in biofilms from the great barrier reef. FEMS Microbiol. Lett. 323, 188–195. 10.1111/j.1574-6968.2011.02374.x, PMID: 22092719

[ref92] Wöhlbrand L. Jacob J. H. Kube M. Mussmann M. Jarling R. Beck A.. (2013). Complete genome, catabolic sub-proteomes and key-metabolites of *Desulfobacula toluolica* Tol2, a marine, aromatic compound-degrading, sulfate-reducing bacterium. Environ. Microbiol. 15, 1334–1355. 10.1111/j.1462-2920.2012.02885.x, PMID: 23088741

[ref93] Yoshida S. Hiraga K. Takehana T. Taniguchi I. Yamaji H. Maeda Y.. (2016). A bacterium that degrades and assimilates poly(ethylene terephthalate). Science 351, 1196–1199. 10.1126/science.aad6359, PMID: 26965627

[ref94] Zecchin S. Mueller R. C. Seifert J. Stingl U. Anantharaman K. von Bergen M. (2018). Rice Paddy *Nitrospirae* carry and express genes related to Sulfate respiration: proposal of the new genus “*Candidatus* Sulfobium”. Appl. Environ. Microbiol. 84, e02224–e02217. 10.1128/AEM.02224-17 29247059 PMC5812927

[ref95] Zettler E. R. Mincer T. J. Amaral-Zettler L. A. (2013). Life in the “plastisphere”: microbial communities on plastic marine debris. Environ. Sci. Technol. 47, 7137–7146. 10.1021/es401288x, PMID: 23745679

[ref96] Zhou J. Bruns M. A. Tiedje J. M. (1996). DNA recovery from soils of diverse composition. Appl. Environ. Microbiol. 62, 316–322. PMID: 8593035 10.1128/aem.62.2.316-322.1996 PMC167800

