# Supplementary material for: Shotgun Metagenomics Reveals the Benthic Microbial Community Response to Plastic and Bioplastic in a Coastal Marine Environment
Source: Front Microbiol. 2019 Jun 7;10:1252. doi: 10.3389/fmicb.2019.01252 (PMC6566015; doi:10.3389/fmicb.2019.01252)
Supplement: TABLE S1 — Library preparation and sequencing information. [file Table_1.DOCX]

**Table S1.** Library preparation and sequencing information.

| **Sample type** | **Final library**  **[DNA] (ng μL^-1^)** | **Average library**  **size (bp)** | **Reads**  **generated** |
| --- | --- | --- | --- |
| Seawater 1 | 4.40 | 523 | 32,009,126 |
| Seawater 2 | 4.48 | 484 | 26,860,030 |
| Seawater 3 | 5.06 | 568 | 31,981,354 |
| Ceramic 1 | 5.40 | 1001 | 30,229,962 |
| Ceramic 2 | 7.12 | 1469 | 30,021,626 |
| Ceramic 3 | 7.28 | 1420 | 29,251,312 |
| PET 1 | 6.66 | 1516 | 36,278,178 |
| PET 2 | 4.80 | 458 | 30,133,310 |
| PET 3 | 5.00 | 1086 | 27,764,482 |
| PHA 1 | 4.44 | 1516 | 29,795,160 |
| PHA 2 | 5.16 | 458 | 29,812,234 |
| PHA 3 | 3.06 | 1086 | 36,522,626 |

Abbreviations: PET, polyethylene terephthalate; PHA, polyhydroxyalkanoate
