# Supplementary material for: Shotgun Metagenomics Reveals the Benthic Microbial Community Response to Plastic and Bioplastic in a Coastal Marine Environment
Source: Front Microbiol. 2019 Jun 7;10:1252. doi: 10.3389/fmicb.2019.01252 (PMC6566015; doi:10.3389/fmicb.2019.01252)
Supplement: TABLE S2 — The top five members from each taxonomic rank based on normalized proportion per sample. Means (n = 3) plus or minus the standard error of mean are reported for each of the taxonomic assignments. [file Table_2.DOCX]

| **Seawater** | **Ceramic** | **PET** | **PHA** |
| --- | --- | --- | --- |
| **Phylum** | | | |
| Proteobacteria  (42.3% ± 0.56) | Proteobacteria  (54.6% ± 1.77) | Proteobacteria  (58.8% ± 1.84) | Proteobacteria  (72.9% ± 1.33) |
| Cyanobacteria  (22.2% ± 0.71) | Bacteroidetes  (11.2% ± 0.08) | Bacteroidetes  (12.3% ± 1.45) | Bacteroidetes  (5.7% ± 0.04) |
| Bacteroidetes  (17.7% ± 0.39) | Cyanobacteria  (6.4% ± 0.62) | Cyanobacteria  (6.2% ± 0.69) | Chloroflexi  (4.2% ± 0.79) |
| Actinobacteria  (8.9% ± 0.41) | Planctomycetes  (4.7% ± 0.09) | Planctomycetes  (3.7% ± 0.07) | Spirochaetes  (3.8% ± 0.44) |
| Planctomycetes  (4.0% ± 0.10) | Verrucomicrobia  (3.2% ± 0.53) | Chloroflexi  (3.7% ± 0.83) | Firmicutes  (1.8% ± 0.11) |
| **Order** | | | |
| Subsection I (Cyanob.)  (20.1% ± 0.71) | Desulfobacterales  (12.4% ± 2.40) | Rhodobacterales  (9.6% ± 1.42) | Desulfobacterales  (36.5% ± 0.30) |
| Oceanospirillales  (10.7% ± 0.22) | Rhodobacterales  (8.0% ± 2.49) | Desulfobacterales  (9.0% ± 2.18) | γ-proteo. *I. sedis*  (4.6% ± 0.35) |
| SAR11 clade  (7.5% ± 0.11) | Xanthomonadales  (4.6% ± 0.41) | Xanthomonadales  (4.7% ± 0.90) | Anaerolineales  (4.0% ± 0.98) |
| Rhodobacterales  (7.4% ± 0.49) | Cellvibrionales  (3.0% ± 0.21) | Chromatiales  (4.1% ± 1.04) | Spirochaetales  (3.8% ± 0.53) |
| Sphingobacteriales  (7.1% ± 0.22) | Cytophagales  (2.9% ± 0.22) | Cellvibrionales  (3.6 ± 0.26) | Chromatiales  (3.0% ± 0.21) |
| **Genus** | | | |
| *Synechococcus*  (14.1% ± 0.52) | uncult. Desulfobacteracae  (4.9% ± 1.13) | uncult. Desulfobacteracae  (3.6% ± 0.88) | uncult. Desulfobacteracae  (8.2% ± 0.14) |
| *Litoricola*  (6.3% ± 0.13) | uncult. Rhodobacteraceae  (3.0% ± 0.92) | uncult. Rhodobacteraceae  (3.0% ± 0.68) | *Desulfobacter*  (6.2% ± 0.82) |
| *Prochlorococcus*  (5.3% ± 0.17) | Desulfobacteracae Sva0081  (2.4% ± 0.35) | uncult. Anaerolineaceae  (2.45 ± 0.72) | *Desulfospira*  (3.0% ± 0.22) |
| uncult. Saprospiraca  (4.6% ± 0.19) | uncult. γ-proteo. *I. sedis*  (1.95% ± 0.20) | Candidatus Thiobios  (2.1% ± 0.47) | uncult. Desulfobulbaceae  (3.0% ± 0.51) |
| Ambiguous SAR11  (4.7% ± 0.10) | uncult. Flammeovirgaceae  (1.94% ± 0.25) | uncult. Flammeovirgaceae  (1.90% ± 0.28) | *Spirochaeta*  (2.3% ± 0.24) |

**Table S2.** The top five members from each taxonomic rank based on normalized proportion per sample. Means (n=3) plus or minus the standard error of mean are reported for each of the taxonomic assignments.

Abbreviations: PET, polyethylene terephthalate; PHA, polyhydroxyalkanoate; uncult., uncultured; Cyanob., Cyanobacteria.
