# Supplementary figures and images for: Shotgun Metagenomics Reveals the Benthic Microbial Community Response to Plastic and Bioplastic in a Coastal Marine Environment
Source: Front Microbiol. 2019 Jun 7;10:1252. doi: 10.3389/fmicb.2019.01252 (PMC6566015; doi:10.3389/fmicb.2019.01252)

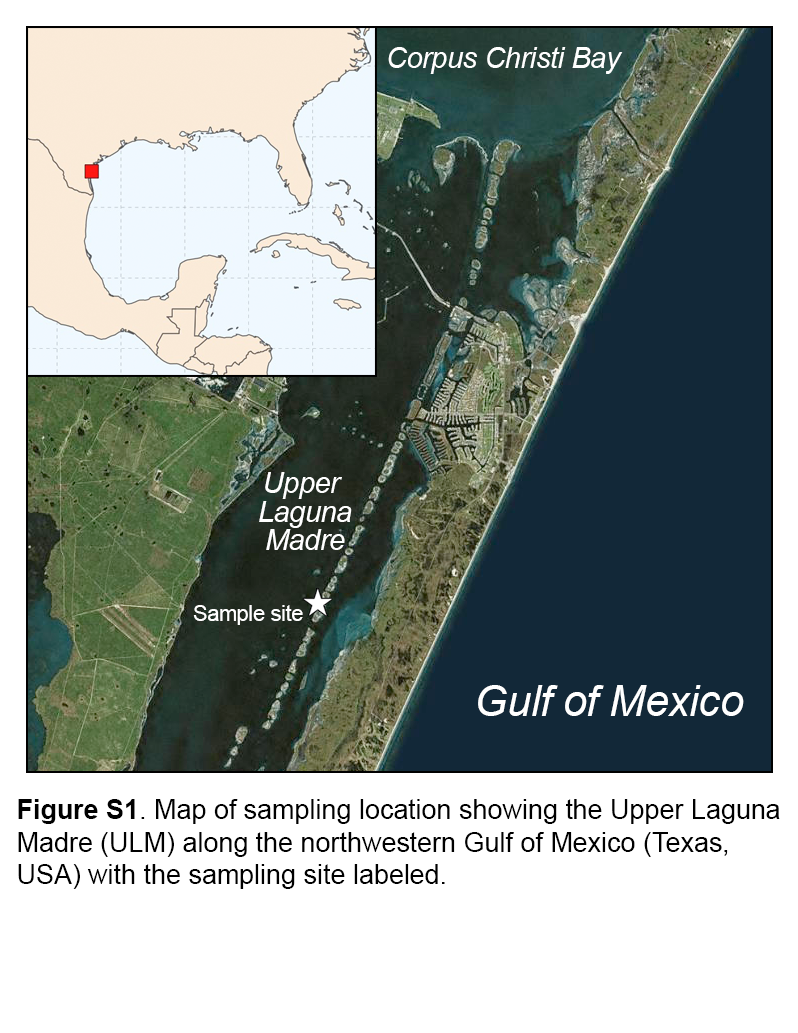

Supplement: FIGURE S1 — Map of sampling location showing the Upper Laguna Madre (ULM) along the northwestern Gulf of Mexico (Texas, USA) with the sampling site labeled. [file Image_1.tiff]

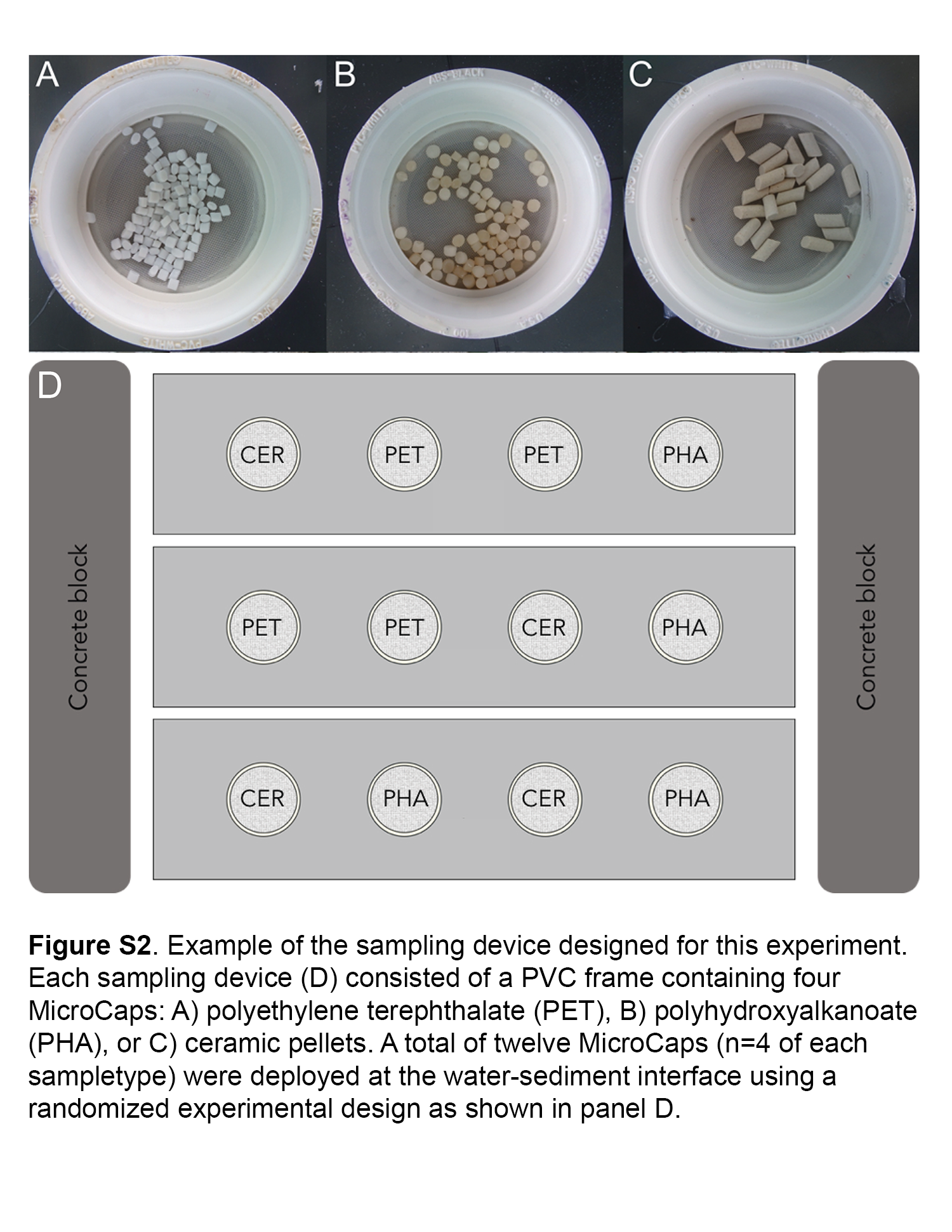

Supplement: FIGURE S2 — Example of the sampling device designed for this experiment. Each sampling device (D) consisted of a PVC frame containing four MicroCaps: (A) polyethylene terephthalate (PET), (B) polyhydroxyalkanoate (PHA), or (C) ceramic pellets. A total of twelve MicroCaps (n = 4 of each sample type) were deployed at the water-sediment interface using a randomized experimental design as shown in panel (D). [file Image_2.tiff]

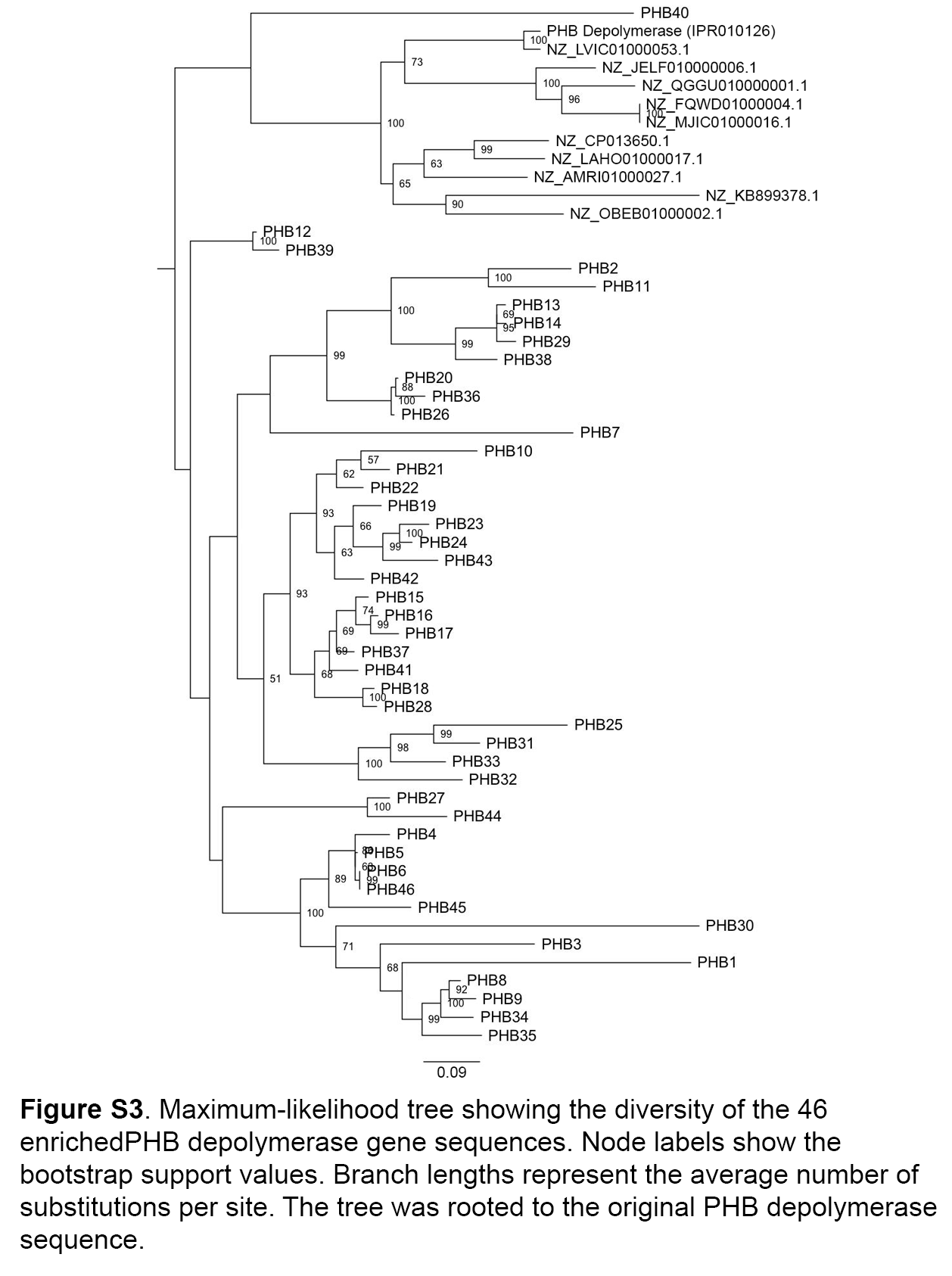

Supplement: FIGURE S3 — Maximum-likelihood tree showing the diversity of the 46 enriched PHB depolymerase gene sequences. Node labels show the bootstrap support values. Branch lengths represent the average number of substitutions per site. The tree was rooted to the original PHB depolymerase sequence. [file Image_3.tiff]

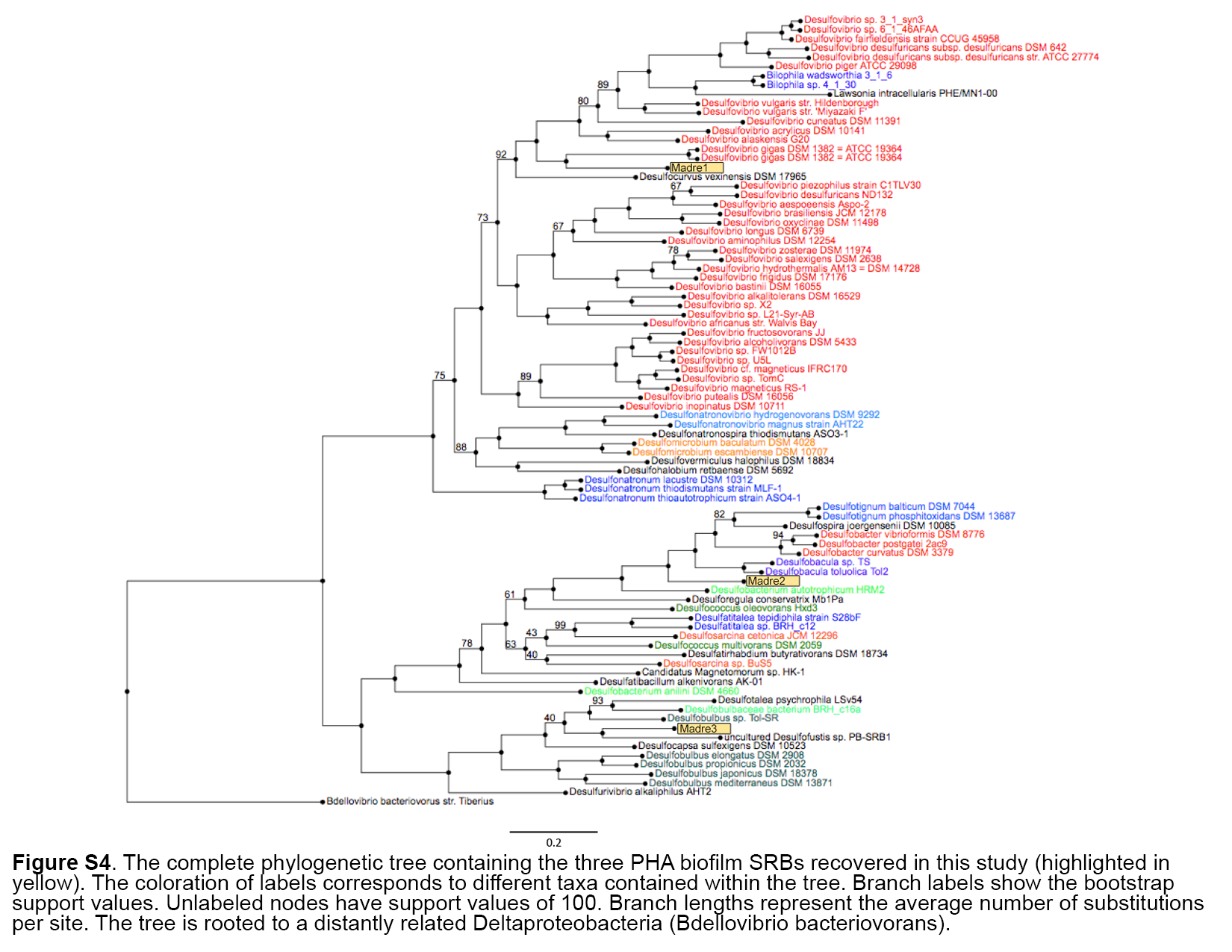

Supplement: FIGURE S4 — The complete phylogenetic tree containing the three PHA biofilm SRBs recovered in this study (highlighted in yellow). The coloration of labels corresponds to different taxa contained within the tree. Branch labels show the bootstrap support values. Unlabeled nodes have support values of 100. Branch lengths represent the average number of substitutions per site. The tree is rooted to a distantly related Deltaproteobacteria (Bdellovibrio bacteriovorans). [file Image_4.tiff]
